# Genome-wide SNP discovery in tetraploid alfalfa using 454 sequencing and high resolution melting analysis

**DOI:** 10.1186/1471-2164-12-350

**Published:** 2011-07-06

**Authors:** Yuanhong Han, Yun Kang, Ivone Torres-Jerez, Foo Cheung, Christopher D Town, Patrick X Zhao, Michael K Udvardi, Maria J Monteros

**Affiliations:** 1 Forage Improvement Division, The Samuel Roberts Noble Foundation, 2510 Sam Noble Parkway, Ardmore, OK, 73401, USA; 2 Plant Biology Division, The Samuel Roberts Noble Foundation, 2510 Sam Noble Parkway, Ardmore, OK, 73401, USA; 3 Center for Human Immunology, Autoimmunity and Inflammation, National Institute of Health, 9000 Rockville Pike, Bethesda, MD 20892, USA; 4 The J. Craig Venter Institute, 9704 Medical Center Drive, Rockville, MD 20850, USA

## Abstract

**Background:**

Single nucleotide polymorphisms (SNPs) are the most common type of sequence variation among plants and are often functionally important. We describe the use of 454 technology and high resolution melting analysis (HRM) for high throughput SNP discovery in tetraploid alfalfa (*Medicago sativa *L.), a species with high economic value but limited genomic resources.

**Results:**

The alfalfa genotypes selected from *M. sativa *subsp. *sativa *var. 'Chilean' and *M. sativa *subsp. *falcata *var. 'Wisfal', which differ in water stress sensitivity, were used to prepare cDNA from tissue of clonally-propagated plants grown under either well-watered or water-stressed conditions, and then pooled for 454 sequencing. Based on 125.2 Mb of raw sequence, a total of 54,216 unique sequences were obtained including 24,144 tentative consensus (TCs) sequences and 30,072 singletons, ranging from 100 bp to 6,662 bp in length, with an average length of 541 bp. We identified 40,661 candidate SNPs distributed throughout the genome. A sample of candidate SNPs were evaluated and validated using high resolution melting (HRM) analysis. A total of 3,491 TCs harboring 20,270 candidate SNPs were located on the *M. truncatula *(MT 3.5.1) chromosomes. Gene Ontology assignments indicate that sequences obtained cover a broad range of GO categories.

**Conclusions:**

We describe an efficient method to identify thousands of SNPs distributed throughout the alfalfa genome covering a broad range of GO categories. Validated SNPs represent valuable molecular marker resources that can be used to enhance marker density in linkage maps, identify potential factors involved in heterosis and genetic variation, and as tools for association mapping and genomic selection in alfalfa.

## Background

DNA pyrosequencing using 454 sequencing technology enables sequencing millions of high-quality DNA bases per sequencing run [1,2]. This method has been used successfully for transcriptome sequencing and identification of single nucleotide polymorphisms (SNP) in many plant species including maize (*Zea mays *L.) [3], sugarcane (*Saccarum *spp.) [4], eucalyptus (*Eucalyptus grandis*) [5], and the model legume and close relative of alfalfa, *M. truncatula *[6]. The *M. truncatula *genome is one of three legume genomes including *Lotus japonicus *and soybean (*Glycine max*) that have been assembled and annotated [7,8]. SNPs are the most common sequence variation among plants and are often functionally important. SNPs can be converted into genetic markers that can be inexpensively assayed using high-throughput approaches [9]. Traditionally, molecular markers have been used to determine genetic relatedness between plant materials, to assist in the identification of novel sources of genetic variation, to study evolutionary relationships, to confirm the pedigree and identity of new varieties, in population structure analysis and association genetics, to locate quantitative trait loci (QTLs) and genes of interest, and for marker-assisted breeding [10]. The value and uses of DNA markers have been shaped in large part by innovations in marker technologies that increase throughput and reduce costs per data point [11]. A large number of molecular markers can be used in high-throughput genotyping platforms in association mapping studies to dissect complex traits and in molecular breeding approaches at the whole genome level [12].

Alfalfa (*Medicago sativa *L.) is one of the most important forage legume species worldwide and the third most valuable crop in the USA ($8 billion per annum). Alfalfa is a high yielding perennial species that requires little or no nitrogen fertilizer because of its ability to carry out symbiotic nitrogen fixation and can be harvested multiple times during the growing season. Therefore, it has been an important component of sustainable agricultural systems for many years and has recently been promoted as a potential bioenergy crop [13]. Cultivated alfalfa is tetraploid (2n = 4x = 32) and displays tetrasomic inheritance [14]. Alfalfa is partially self-incompatible and populations are extremely polymorphic due to their high degree of outcrossing. Inbreeding severely depresses plant vigor and fertility in tetraploid alfalfa due to the loss of complementary gene interactions [15,16], preventing the development of inbred lines. A significant level of sequence conservation was reported between alfalfa and *M. truncatula *[17] allowing estimates of marker colinearity between the two species [18]. Simple sequence repeat (SSR) markers developed from *M. truncatula *and some alfalfa sequences are currently available [19-21]. Alfalfa like many other crop species, lacks validated SNP markers which are required in large numbers for map-based gene isolation, association genetics and genomic selection approaches [10].

High-resolution melting (HRM) curve analysis has proven to be a highly sensitive method for mutation discovery and SNP genotyping [22]. Nucleic acid melting is tracked by monitoring the fluorescence of the samples across a defined temperature range generating high-resolution melting profiles that are used to identify the presence of sequence variation within the amplicon [23]. Base-pair mismatches shift the stability of a duplex by varying amounts depending on the particular mismatch, the mismatch position, and neighboring base pairs [24]. HRM was successfully implemented to assay SNP variation in diploid and tetraploid alfalfa [25].

We describe the generation of thousands of alfalfa ESTs (expressed sequence tags) obtained using 454 sequencing technology and the first iteration of genome-wide SNP identification in tetraploid alfalfa, a non-model plant species that currently lacks a sequenced genome. A sample of candidate SNPs were experimentally validated using a HRM platform.

## Results

### Sequence assembly and annotation

**Figure 1:**
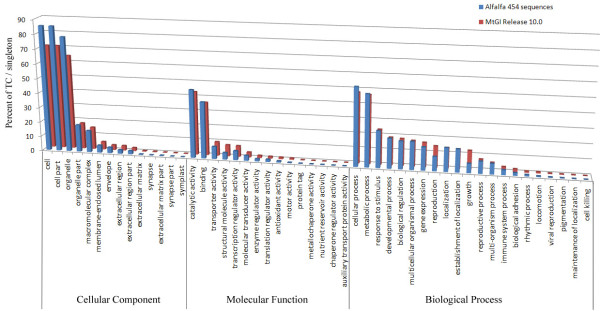
**Comparative distribution of gene ontology assignment of sequences derived from 454 sequencing of cDNA from *M. sativa *and those in the *M. truncatula *gene index (MtGI release 10.0)**. Assignment is expressed as percent of singleton/TC allocated to functional groups within each of three broad categories.

Root and shoot samples were collected during progressively developing water stress from clonal propagules of each alfalfa genotype. RNA was purified and a pooled sample for each genotype was used to construct 454 Titanium cDNA libraries. The two libraries were sequenced on separate 454 half-plates that generated (after trimming and removal of reads less than 100 bp) 155,120 reads with a total length of 57.5 Mb from genotype Chilean and 177,871 reads with a total length of 67.6 Mb from genotype Wisfal (Table 1). The raw sequences were clustered and assembled separately for each genotype and also as a combined set using TGICL clustering utility [26]. The combined sequence assembly with the two genotypes resulted in 24,144 TCs and 30,072 singletons ranging from 100 to 6,662 bases, with an average length of 541 bases. Alfalfa transcriptome sequences were submitted to the short read database and transcript assembly database in GenBank (http://www.ncbi.nlm.nih.gov) and were assigned the accession numbers [Genbank: JL866457-JL881209], [Genebank: JL881210-JL898333], [Genbank: SRX040822], and [GenBank: SRX040823], respectively for Wisfal short reads, Chilean short reads, Chilean assembly, and Wisfal assembly. For the purposes of annotation, the combined alfalfa transcript assemblies were located on the *Medicago truncatula *Gene Index (MtGI) Release 10.0 maintained by the Dana Farber Cancer Institute (http://compbio.dfci.harvard.edu/cgi-bin/tgi/gimain.pl?gudb=medicago). A total of 41,350 alfalfa unique sequences (76%) had a blastn hit to 24,358 MtGI sequences (cut off E value 1e-^10^). For the 41,350 sequences with hits in the MtGI database, Gene Ontology (GO) categories of their best match were assigned to each sequence. The proportion of alfalfa 454 sequences in each category follows the same general trend as the MtGI (Figure 1), indicating sequence coverage of a broad range of genes in the alfalfa 454 sequences.

**Table 1:** Alfalfa sequence length distribution pre-and post-assembly

Pre-assembly	Chilean	Wisfal	Combined
Total base count (bp)	57,563,286	67,698,210	125,261,496
Sequence count	155,120	177,871	332,991

100-200 bp	13,881	10,380	24,261
200-300 bp	23,360	22,388	45,748
300-400 bp	40,842	55,262	96,104
400-500 bp	65,222	79,475	144,697
500-600 bp	11,806	10,362	22,168
600-700 bp	9	4	13

Minimum length (bp)	100	100	100
Maximum length (bp)	621	636	636
Average length (bp)	374	382	379

Post-assembly	Chilean	Wisfal	Combined

Total base count (bp)	18,581,045	20,813,330	29,353,134
TC count	14,815	17,178	24,144
Singleton count	22,918	22,990	30,072

100-500 bp	34,794	25,574	26,941
501-1000 bp	14,445	9,940	10,385
1001-1500 bp	3,174	1,573	1,976
1501-2000 bp	1,155	454	604
> 2000 bp	648	192	262

Minimum length (bp)	100	100	100
Maximum length (bp)	4,974	5,124	6662
Average length (bp)	492	518	541

### Alignment of alfalfa sequences to the M. truncatula genome

**Figure 2:**
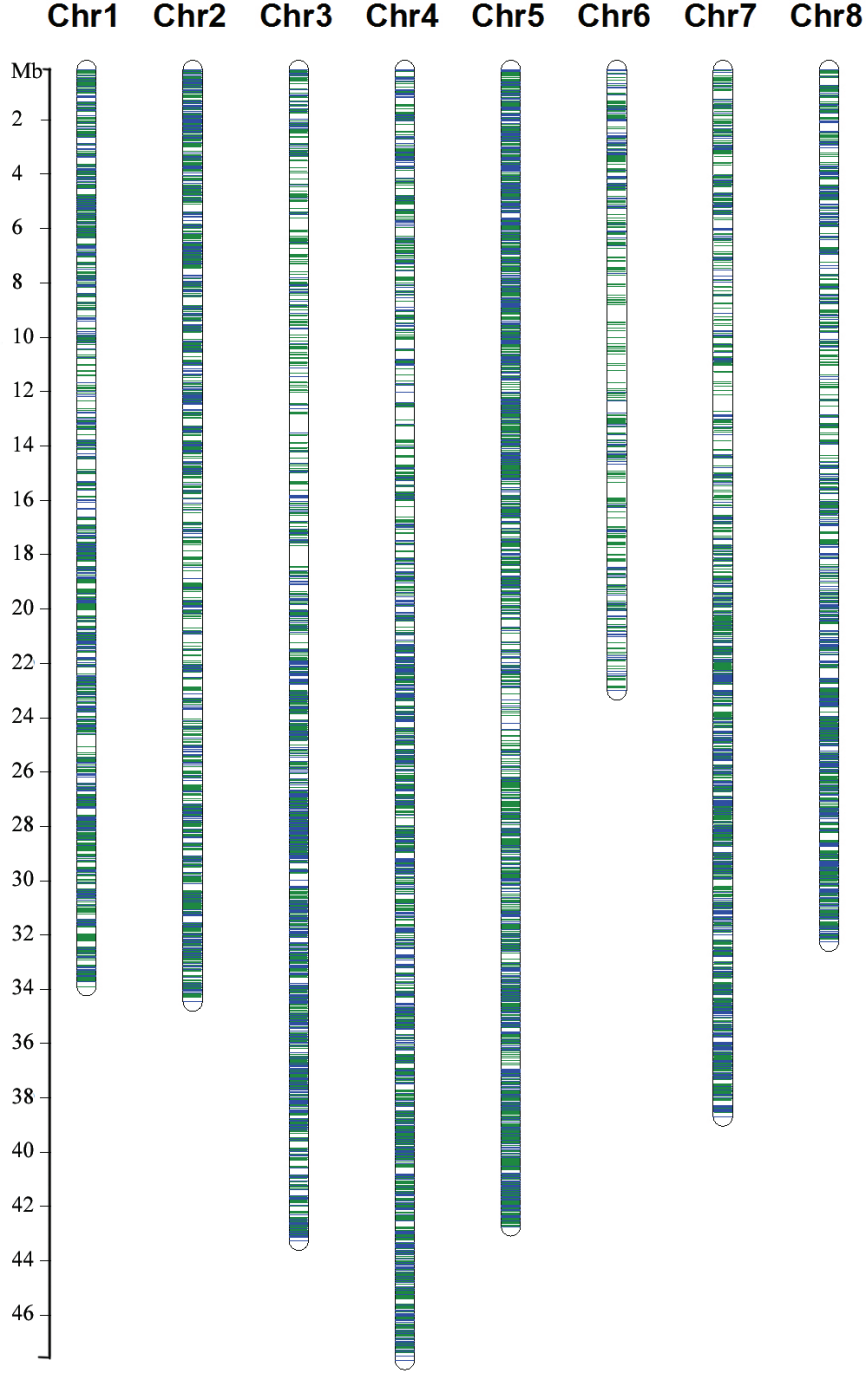
**Alfalfa sequences mapped to MT 3.5 pseudomolecules with a threshold of 95% identity and 90% coverage**. Blue lines represent alfalfa sequences without candidate SNPs and green lines represent alfalfa sequences containing candidate SNPs.

The available *M. truncatula *genome sequence was used as a scaffold to align the alfalfa transcript sequences. Under stringent conditions using Blat, including a threshold of 95% identity and 90% coverage (Figure 2), 20,067 (10,911 TCs and 9,156 singletons) unique sequences (37%) were mapped to the MT 3.5.1 genome sequence assembly. Among these, a total of 20,270 candidate SNPs were identified in 3,491 TCs mapped to the *M. truncatula *genome (Table 2) and their likely map positions inferred. To identify the number of distinct loci represented in the alfalfa transcript assemblies, they were searched against the *M. truncatula *v 3.5 pseudomolecules annotation. At the nucleotide level, 32,606 (60%) of the alfalfa 454 sequences had a blastn (cut off E value 1e^-10^) hit with 15,932 *M. truncatula *CDS. We essentially obtained the same result at the amino acid level, in which 35,791 (66%) of the alfalfa 454 sequences had a blastx hit (cut off E value 10^-5^) to 15,870 *M. truncatula *predicted proteins.

**Table 2:** Summary of candidate SNP containing assemblies and their location on the MT3.5 chromosomes

	Singleton	TC	TC with SNPs	Number of SNPs
Total	30072	24144	7342	40661
Mapped to Mt. 3.5	9156	10911	3491	20270

Chr1	995	1274	388	2236
Chr2	1102	1224	404	2497
Chr3	1208	1548	476	2551
Chr4	1393	1775	591	3592
Chr5	1522	1820	577	3162
Chr6	428	343	122	867
Chr7	1113	1411	430	2310
Chr8	1018	1089	372	2319
Chr0	377	427	131	736

When combined with the MtGI results, 46,086 (85%) alfalfa sequences had a blastn or blastx hit in MtGI and/or MT3.5 databases. Of the remaining 8,130 alfalfa sequences, 992 of them had a blastx hit (cut off E value 1e^-5^) with 763 proteins in the soybean Glyma1.0 high confidence gene protein database [27] and 732 alfalfa sequences had a blastx hit (cut off E value 1e^-5^) with 572 *L. japonicus *proteins [28] further supporting the notion of shared sequences among legumes. Only 12 of the remaining 7,057 alfalfa sequences without a match in any of the aforementioned databases had a hit in the Arabidopsis TAIR 9.0 protein database (cut off E value 1e^-5^). The remaining 7,045 sequences represent a total length of 2.3 Mb (8% of the total sequences post-assembly), did not have a significant match when blasted against the NCBI non-redundant nucleotide database suggesting they may be miss-assembled or novel sequences.

### SNP discovery and validation

**Figure 3:**
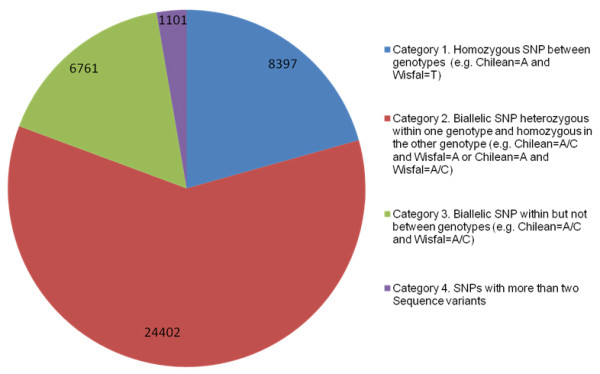
**Categories of candidate SNPs identified through alignment of *M. sativa *subs. *sativa *var 'Chilean' vs. *M. sativa *subsp. *falcata *var 'Wisfal' 454 sequences**.

Candidate SNPs were called based on sequence alignments within and between the Chilean and Wisfal genotypes. A total of 40,661 candidate SNPs were identified in 7,342 TCs within or between the two genotypes. The SNPs were assigned to four Categories based on their allelic composition (Figure 3). Candidate SNPs in the first three Categories have only two variants and include 97% of all candidate SNPs identified. Category 1 SNPs are between the two genotypes and homozygous within each genotype (e.g. Chilean = A, and Wisfal = C). Category 2 SNPs are heterozygous within one genotype and homozygous in the other (e.g. Chilean = A/C and Wisfal = A, or Chilean = A and Wisfal = A/C). Category 2 is the most abundant type of SNPs and constitutes 60% of all candidate SNPs; Category 1 is the second most abundant type with 21% of all candidate SNPs. SNPs in Category 3 (16%) are biallelic and heterozygous within each genotype (e.g. Chilean = A/C and Wisfal = A/C). Category 4 SNPs have more than two variants (e.g. Chilean = A/C/G or/and Wisfal = A/C/T) and account for only 3% of all candidate SNPs identified. We focused on validating Category 2 SNPs due to their suitability for mapping in tetraploid alfalfa based on their segregation patterns in this population. Category 1 SNPs are heterozygous in all F_1 _individuals from the mapping population resulting from a cross between two homozygous genotypes and thus not suitable for mapping. In this study, candidate SNPs represented in Categories 3 and 4 were not considered for validation or mapping due to the complexity of their segregation patterns in this autotetraploid outcrossing species.

**Figure 4:**
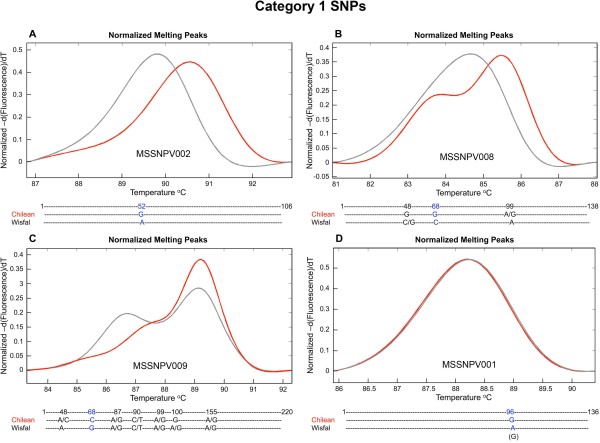
**High resolution melting peaks and amplicon sequences from Category 1 candidate SNPs between *M. sativa *subs. *sativa *var 'Chilean' (red) and *M. sativa *subsp. *falcata *var 'Wisfal' (gray)**. SNPs predicted by 454 sequencing are shown in blue and additional SNPs identified through amplicon sequencing are shown in black. A) Primers SNP MSSNPV002 confirms the presence of a single SNP at position 52 (Chilean = G, Wisfal = A) in a 106 bp amplicon. B) Primers SNP MSSNPV008 confirms the presence of SNP at position 68 (Chilean = G, Wisfal = C), and additional SNPs at position 48 (Chilean = G, Wisfal = C/G) and position 99 (Chilean = A/G, Wisfal = A) in a 138 bp amplicon. C) Validated candidate SNP MSSNPV009 at position 68 (Chilean = C, Wisfal = G), and additional SNPs at position 48 (Chilean = A/C, Wisfal = A), position 87 (Chilean = A/G, Wisfal = A/G), position 90 (Chilean = C/T, Wisfal = C/T), position 99 (Chilean = A/G, Wisfal = A/G), position 100 (Chilean = G, Wisfal = A/G), and position 155 (Chilean = A/G, Wisfal = A/G) in a 220 bp amplicon. D) Non-validated candidate SNP MSSNPV001 (Chilean = G, Wisfal = G) in a 136 bp amplicon.

**Figure 5:**
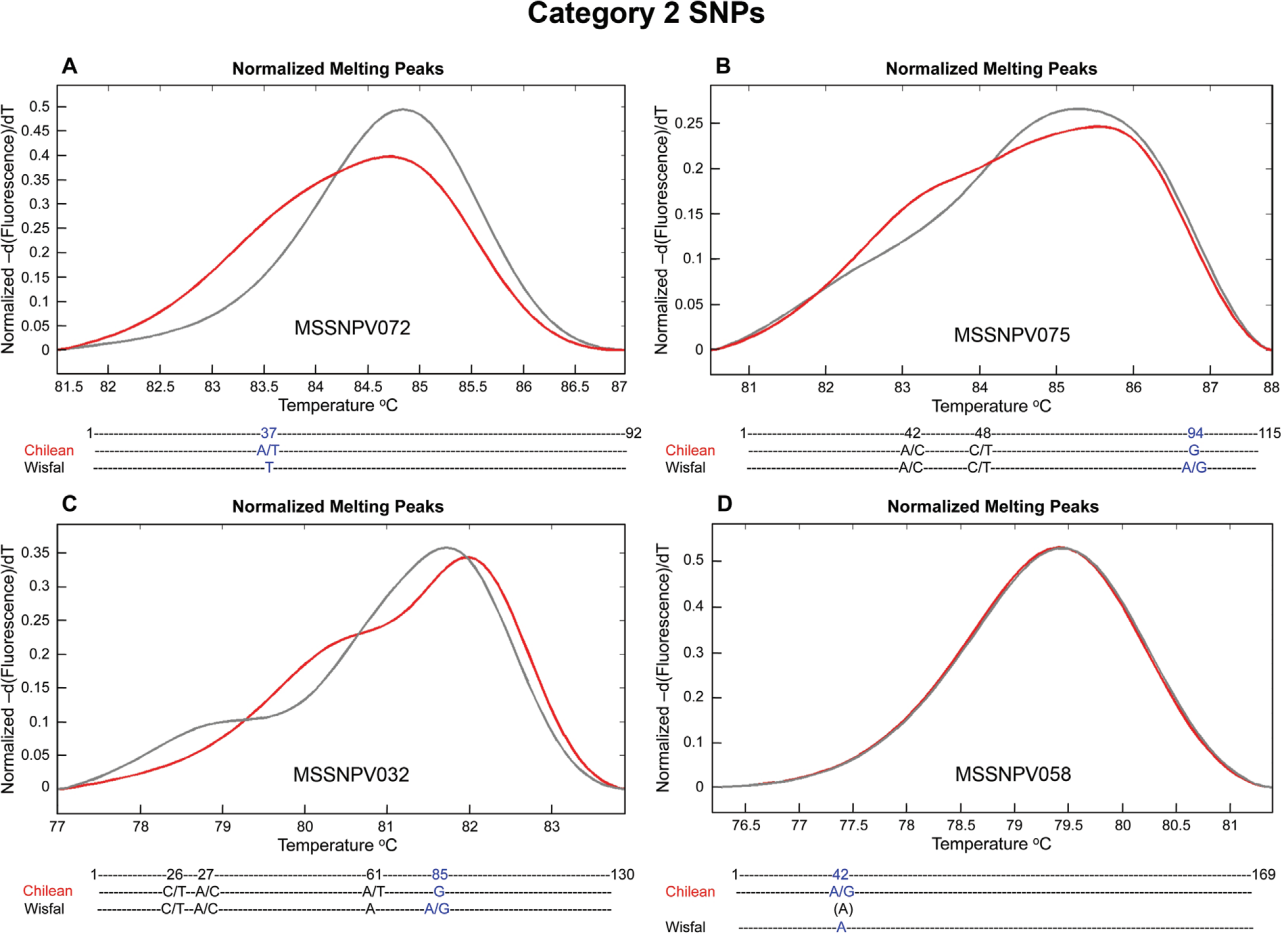
**High resolution melting peaks and amplicon sequences from Category 2 candidate SNPs and the corresponding amplicon sequences between *M. sativa *subs. *sativa *var 'Chilean' (red) and *M. sativa *subsp. *falcata *var 'Wisfal' (gray)**. SNPs predicted by 454 sequencing are shown in blue and additional SNPs identified through amplicon sequencing are shown in black. A) Primers SNP MSSNPV072 confirms the presence of a single SNP at position 37 (Chilean = A/T, Wisfal = T) in a 92 bp amplicon. B) Primers SNP MSSNPV075 confirms SNP at position 94 (Chilean = G, Wisfal = A/G), and additional SNPs at position 42 (Chilean = A/C, Wisfal = A/C), and position 48 (Chilean = C/T, Wisfal = C/T), in a 115 bp amplicon. C) Validated candidate SNP MSCWSNP032 at position 85 (Chilean = G, Wisfal = A/G), and additional SNPs at positions 26 and 27 (Chilean = C/T and A/C, and Wisfal = C/T and A/C, respectively), and position 61 (Chilean = A/T and Wisfal = A), in a 130 bp amplicon). D) Non-validated candidate SNP MSCWSNP058 (Chilean = A and Wisfal = A) in a 169 bp amplicon.

A subset of 192 candidate SNPs (MSSNPV001 to MSSNPV192) identified in the alfalfa transcriptome were used to design primer pairs (Additional File 1) for SNP validation through PCR amplification and HRM analysis. The majority of SNP primers (176) were designed to target Category 2 candidate SNPs and 16 primer pairs targeted Category 1 candidate SNPs (Additional File 2). A Category 1 SNP is homozygous within each genotype but different between the two genotypes. The anticipated HRM profile for a Category 1 SNP includes two different and distinct HRM peaks, each of which corresponds to a different homozygous allelic state. Category 1 SNP marker MSSNPV002 (Figure 4A), results in a melting peak for Wisfal (AAAA, shown in black) and a melting peak for Chilean (GGGG, shown in red). Two of the 16 Category 1 candidate SNPs were not validated and resulted in overlapping melting peaks between the two genotypes, as shown for MSSNPV001 (Figure 4D). The melting curve peak from Category 2 SNP MSSNPV072 corresponding to Chilean, the heterozygous genotype, is shorter and wider than the peak corresponding to Wisfal, the homozygous genotype (Figure 5A). The HRM profiles from the remaining 13 Category 1 candidate SNPs were complex, indicating the existence of additional SNPs within the amplicon sequence that were not predicted by 454 sequencing (Figure 4B and 4C). Results from the HRM evaluation of the 176 Category 2 candidate SNPs indicate that 119 of them displayed the anticipated Category 2 SNP profile (Figure 5A), 49 showed complex profiles indicating the presence of additional SNPs not predicted based on the 454 sequences (Figure 5B and 5C), and eight candidate SNP were not validated (Figure 5D) based on overlapping melting curves and amplicon sequencing (Figure 5D). Sequencing of the PCR amplicon obtained from MSSNPV072 confirmed a single SNP at position 37 between the two genotypes (Figure 5A). A total of 120 SNPs were validated, while 62 SNP primers (32.3%) resulted in the identification of additional SNPs. For example, the predicted Category 1 SNP for MSSNPV008 at position 68 (G vs. C) was confirmed by sequencing and additional SNPs were identified at position 48 and position 99 (Figure 4B). The predicted Category 2 SNP at position 94 for MSSNPV075 was confirmed, and additional SNPs were identified at position 42 and position 48 (Figure 5B). In the case of SNP MSSNPV032, the predicted SNP at position 85 was confirmed, and three additional SNPs were also detected (Figure 5C). Variation in depth of sequence coverage was observed for the SNP-containing sequences between the two genotypes. Both genotypes were represented in at least 46 sequence reads in the case of validated SNP MSSNPV002 (data not shown). In contrast, candidate SNP MSSNPV001 had less than five sequence reads representing each genotype. Overall, validated candidate SNPs had higher depth of 454 sequence coverage than non-validated SNPs in both Category 1 and Category 2 SNPs.

## Discussion

SNP markers have a broad range of applications and a large number of markers are needed to implement association genetics approaches and genomic selection in plants with low levels of LD [12,29]. Large scale SNP discovery efforts in a wide range of crop plants are currently limited to a few species. Previous SNP discovery approaches in other species include sequence comparisons between the Arabidopsis ecotypes Landsberg and Columbia [30] and subspecies of rice [31]. Although alfalfa is the most widely cultivated forage legume, it lacks genomics resource development compared to other crop species, partly because it is an autotetraploid, perennial outcrossing species. Thus, a whole genome SNP discovery effort is among the most critical endeavors to develop the necessary tools and integrate molecular breeding approaches in alfalfa. In this study, we utilized 454 sequencing for genome-wide SNP discovery between and within two genotypes from *M. sativa *subsp. *sativa *(Chilean) and *M. sativa *subsp. *falcata *(Wisfal), which have contrasting phenotypes for water stress tolerance. Sequencing and SNP discovery based on transcriptome sequencing of water stressed plants increased the likelihood of having sequence coverage in genes relevant to water-stress and identification of allelic differences in these genes between the two alfalfa genotypes evaluated in this study.

### Sequence assembly and alignment to *M. truncatula*

The close phylogenetic relationship between *M. truncatula *and alfalfa, same basic chromosome number (x = 8), high degree of sequence similarity and marker co-linearity [17,18] facilitated using the *M. truncatula *genome sequence as a scaffold to align alfalfa transcriptome sequences. Despite the size and complexity of the alfalfa genome, alignments of alfalfa 454 sequences throughout the *M. truncatula *genome were apparent (Figure 2). The *M. truncatula *genome sequence project targeted the euchromatic region and available data indicate that 66% of the genes captured in the current pseudomolecules and 24% of the genes in *M. truncatula *captured in the fragmented Illumina assemblies are found within the euchromatin. Therefore, the *M. truncatula *genome provides a valuable resource for gene annotation of alfalfa sequences. The identification of some alfalfa sequences without alignment to the *M. truncatula *genome is not unexpected considering that *M. truncatula *is a selfing, annual diploid species and alfalfa is an outcrossing, perennial tetraploid species with a considerably larger genome. Additionally, non-alignment of alfalfa sequences to the *M. truncatula *genome could occur in regions where there have been authentic genome rearrangements between the two species. We demonstrated the feasibility of using genomic resources from model species to quickly assemble transcriptome sequences from related species. These alfalfa resources can also facilitate opportunities to further understand legume genome structure and evolution.

### SNP discovery and validation

454 sequencing is a high-throughput approach to identify sequence variation and was utilized successfully here to identify SNP variation in tetraploid alfalfa. Alfalfa is genetically very diverse and the existence of heterosis between *M. sativa *subsp. *sativa *and *M. sativa *subsp. *falcata *was previously reported [32,33]. Because Chilean and Wisfal are highly heterozygous outcrossing lines, transcriptome sequencing of alfalfa genotypes enabled the identification of gene-associated SNPs within and between genotypes covering a range of gene functions and location in the genome. Reports that 454 sequencing technology may be susceptible to indel-type errors [1] prompted us to consider only base substitutions (i.e. SNPs) with stringent SNP prediction criteria.

The HRM platform proved to be a suitable method for validation and genotyping of a large number of candidate SNPs. The HRM melting curve profiles enables identification of homozygous and heterozygous SNP genotypes [34]. We report a 62.5% rate of validation of single candidate SNPs (120 out of the 192) indicating that transcriptome sequencing of a crop legume with economic value and assembly of those transcript sequences using the genome of a closely related model species is a viable strategy for genome-wide SNP discovery efforts. Studies in eucalyptus reported a 83% rate of validation from the candidate SNPs [5]. Additional SNP in alfalfa were detected using HRM that were not predicted based on the bioinformatics analysis from the 454 sequences. These findings were partly due to our efforts to minimize the false discovery rate in sequences with low depth of sequence coverage. Therefore, the challenge for SNP discovery using 454 sequences in a highly heterozygous species such as alfalfa resides in limitations from the depth of sequence coverage rather than sequencing errors. In contrast to a diploid species, tetraploid species have four homologous chromosomes and therefore deeper sequence coverage is needed to accurately ascertain the SNP variation within and among genotypes. Although an increase in the rate of SNP prediction can be obtained using less stringent SNP parameters, it also increases the likelihood of false positive SNP calls. The discrepancy between SNP detection and validation could also be the result of preferential allele expression or allele silencing in the pooled cDNA sample that was used because the initial SNP identification was based on cDNA sequencing whereas validation was made using PCR fragments amplified from genomic DNA. The identified SNPs complement our current work identifying genomic regions associated with water stress tolerance in backcross populations developed from the same two genotypes used for 454 sequencing.

Previous efforts to identify sequence polymorphism in alfalfa include array hybridizations that identify single feature polymorphisms (SFP) using *M. truncatula *Affy Chips [35]. The approach described here enables the discovery of SNPs within genes that may not be present on an array developed for the model *M. truncatula*. Additionally, SNP discovery efforts based on transcriptome sequencing in a crop species may result in the identification of SNPs with functional relevance to agriculturally important traits such as persistence, likely not present in an annual model system.

The large number of candidate SNPs with potential functional relevance identified in this study provides a valuable tool for comparative genomic studies between *M. truncatula *and alfalfa, and potentially to other forage legume species including white and red clover. Evaluations of SNP variability, frequency, and distribution, as well as estimates of linkage disequilibrium in alfalfa are now attainable research targets. Applications of crop-related SNP discovery efforts include genetic diversity analysis, increasing the marker density of genetic maps to support high-resolution association and linkage mapping of target traits, facilitation of future genome assembly of this highly complex genome, integration of genetic and physical maps, and increasing the feasibility of integrating marker-dense molecular breeding approaches such as genomic selection in this important forage legume.

## Conclusions

In order to enhance crop productivity, we need to improve our understanding of the genes involved in plant growth, development, and adaptation to a changing environment including suboptimal soil conditions and biotic and abiotic stress conditions. The 454 EST-based SNP discovery and validation pipeline described here focused on two tetraploid alfalfa subspecies with contrasting water stress tolerance provides a platform for the discovery of agriculturally-relevant genes and corresponding SNPs. The availability of a large number of validated SNPs is a valuable molecular marker resource for genetics, high-resolution linkage and association mapping, and molecular breeding approaches using genomic selection, for practical and agriculturally-relevant improvements in alfalfa which are not feasible using model systems.

## Methods

### Plant materials

Two tetraploid alfalfa genotypes (sampled from *M. sativa *subsp. *sativa *var. 'Chilean' and *M. sativa *subsp. *falcata *var. 'Wisfal' [36] were clonally propagated and grown in metromix and sand mixture (4:1 v:v). Plant tissue was sampled at seven time points during a progressively developing water deficit including: well-watered, three, seven, nine, 11 and 14 days after withholding water, and one-day re-watering on day 15. Roots and shoots were sampled separately. Two to four clonal propagules of each genotype were sampled at each time point. For each genotype, cDNA was prepared from shoot and root tissue of each clonally propagated sample grown under either well-watered or water-stressed conditions, and then equal amounts of cDNA from each sampling point were pooled for sequencing using 454 sequencing technology.

### cDNA preparation and sequencing

Total RNA was extracted using TRIzol^® ^(Invitrogen, Carlsbad, CA) following the manufacturer's recommendations, digested with DNAseI (Ambion, Austin, TX), column purified with RNeasy MinElute Cleanup kit (Qiagen, Valencia, CA) and quantified using a NanoDrop Spectrophotometer ND-100 (NanoDrop Technologies, Willington, DE). Total RNA integrity was assessed using a Bioanalyzer 2100 (Agilent, Santa Clara, CA). Messenger RNA (mRNA) isolation was performed using Poly(A)Purist(tm) MAG (Magnetic mRNA Purification Kit, Ambion, Austin, TX). For the first strand cDNA synthesis, three microgram of mRNA was used with Oligo (dT)_12-18 _and Superscript II Reverse Transcriptase followed by a second cDNA synthesis (dscDNA) using Superscript First and Double Stranded cDNA synthesis Kit (Invitrogen, Carlsbad, CA). Double stranded cDNA from roots and shoots from the same genotype was pooled and deep sequenced using Titanium 454 technology at the J. Craig Venter Institute (JCVI) with each genotype occupying half of a 454 sequencing plate.

### Sequence analysis

The raw sequences were assembled for each genotype separately and then combined (Table 1) using TGICL clustering utility [24] with The Paracel Transcript Assembler instead of CAP3 and a modified version of the TIGR Gene Index pipeline. The blast searches were performed using TCs and singletons from the assembly with the two genotypes combined (Table 1). All TCs and singletons were blasted against the MtGI 10.0 database using Blastn with e^-10 ^as a cut-off expected value. The TCs and singletons were assigned to the GO categories based on their top hit.

### SNP discovery and validation

Candidate SNP calls were predicted using Perl scripts to parse the transcript assembly files and requiring a minimum coverage of at least two 454 reads for each form of the SNP at the variant position. A confidence score was generated based on the percent of nucleotides matching the surrounding regions of the location of the SNP. Low confidence regions with less than 90% identity where a SNP was called were not included in the final SNP predictions. SNPs were quality controlled by remapping the 454 reads back onto the assembly and checked against the ACE alignment files. Indels were ignored and the process was biased towards getting good quality SNPs vs. getting all the SNPs at the expense of false SNP calls. Primer3 was used to design primers targeting candidate SNPs. Criteria for primer design include a predicted annealing temperature (Tm) of 59°C to 61°C, primer length ranging between 18-24 bp and PCR amplicon lengths of 40 to 200 bp. All PCR reactions were performed in 384-well plates using a 9700 Thermal Cycler (Applied Biosystems, Foster City, CA, USA) using a total volume of 5 mL per well. The PCR reaction mixture consisted of 5 ng of genomic DNA, 0.25 μ*M *of forward and reverse primer, 1X LightScanner High Sensitivity Master Mix (Idaho Technologies, Salt Lake, UT, USA) and 10 mL mineral oil. After an initial denaturation step of 2 min at 95°C, 45 PCR cycles were performed with 30 s of denaturation at 94°C and 30 s at the target annealing temperature, followed by a final hold at 4°C. Samples were then transferred to a LightScanner 384-well system (Idaho Technologies, Salt Lake, UT) and a melting cycle was performed by increasing the temperature at 0.1°C s^-1 ^from 56 to 95°C. Melting data was analyzed and visualized with the LightScanner Software with CALL-IT 2.0 (Idaho Technologies, Salt Lake, UT) using the small amplicon module.

### Re-sequencing PCR amplicons

The PCR amplicons from twenty candidate SNP primers were cleaned using Exo/SAP-IT PCR product cleanup kit following the manufacturer's instructions (Affymetrix, Inc., Cleveland, OH) and directly sequenced using a BigDye Terminator v3.1 Cycle Sequencing Kit (Applied Biosystems, Foster City, CA, USA) using both forward and reverse primers. The amplicons were analyzed using an ABI 3730 automated sequencer (PE-ABI, Foster City, CA). The sequence data was analyzed and aligned for SNP discovery with Sequencher 4.8 (Gene Codes).

## Authors' contributions

YH identified candidate SNPs, performed SNP validation, and drafted the manuscript. YK and ITJ collected the samples, extracted RNA and prepared cDNA for sequencing. FC and CDT performed 454 sequencing, data assembly, SNP predictions and alignment of reads to the *M. truncatula *genome. PXZ assisted in the bioinformatics analysis of sequence data and SNP identification. MU supervised sample preparation and sequencing. MM managed the overall project and drafted the manuscript. All authors have read and approved the final manuscript.

## Supplementary Material

Additional file 1 **Characteristics of 192 SNP primer pairs developed using alfalfa 454 sequences**. The file contains the primer name, SNP category, amplicon size, Tm, and primer sequences. Click here for file

Additional file 2 **Summary of validation results of 192 SNP in alfalfa using HRM analysis**. The file contains the SNP category, number of SNP, and the SNP validation status. Click here for file
