# Supplementary material for: Genome-wide SNP discovery in tetraploid alfalfa using 454 sequencing and high resolution melting analysis
Source: BMC Genomics. 2011 Jul 6;12:350. doi: 10.1186/1471-2164-12-350 (PMC3154875; doi:10.1186/1471-2164-12-350)
Supplement: Additional file 2 — Summary of validation results of 192 SNP in alfalfa using HRM analysis. The file contains the SNP category, number of SNP, and the SNP validation status. [file 1471-2164-12-350-S2.DOC]

**Additional File 2**. Summary of validation results of 192 SNPs using HRM analysis

|  |  | Validation status | | |
| --- | --- | --- | --- | --- |
| SNP Category | No. of SNPs | Validated SNP | Validated SNP + additional SNP | Non-validated  SNP |
| Category 1 | 16 | 1 (6.2%) | 13 (81.3%) | 2 (12.5%) |
| Category 2 | 176 | 119 (67.7%) | 49 (27.8%) | 8 (4.5%) |
| Total | 192 | 120 (62.5%) | 62 (32.3%) | 10 (5.2%) |
